# Individual differences and moderating participant characteristics in the effect of reducing portion size on meal energy intake: Pooled analysis of three randomized controlled trials

**DOI:** 10.1016/j.appet.2020.105047

**Published:** 2021-04-01

**Authors:** Eric Robinson, Ashleigh Haynes

**Affiliations:** a Department of Psychological Sciences, University of Liverpool, L69 7ZA, UK; b Centre for Behavioural Research in Cancer, Cancer Council Victoria, Melbourne, Australia

**Keywords:** Portion size, Individual differences, Energy intake, Eating behaviour, BMI (body mass index), DEBQ (Dutch eating behaviour questionnaire)

## Abstract

Portion size impacts on the amount of energy consumed during a meal. However, research findings on participant characteristics that moderate the effect of portion size on energy intake are mixed. Using data pooled across three randomized control trials, we examined the impact of reducing meal portion size on meal energy intake in 111 adult participants varying in sex (55 M, 56 F), body weight (BMI range = 19–42) and a broad range of participant characteristics, including usual portion size, restrained, emotional and external eating, satiety responsiveness, plate clearing tendencies, concerns about wasting food and self-control. In each trial, a repeated-measures design was used and participants consumed three ad-libitum lunchtime meals differing in portion size; large-normal portion size condition (100%) vs. small-normal portion size condition (~ 75%) vs. smaller than normal portion size condition (~ 50%). In mixed ANOVAs, we did not find convincing evidence that any participant characteristic reliably moderated the impact that reducing portion size had on energy intake. For the majority of participants energy intake decreased when portion size was reduced and it was more common for participants to consistently reduce their energy intake than consume a similar amount when portion size was reduced. We also found little evidence that a sub-group of participants existed whose energy intake was consistently resistant to portion size reductions. Portion size may be a universal driver of energy intake, as reducing meal portion size appears to decrease meal energy intake among most people. Food portion downsizing may therefore be an equitable intervention approach to reducing population level energy intake.

## 1 Introduction

Increasing the portion size of food served at a meal has been to shown to have a causal influence on the amount of energy consumed during that meal (Rolls, Morris, & Roe, 2002). Moreover, there is evidence that the effect food portion size has on energy intake is sustained over several days (French et al., 2014; Rolls, Roe, & Meengs, 2007). Because of this, reducing food portion sizes has been identified as a potential intervention strategy to decrease population level energy intake in order to address obesity (Marteau, Hollands, Shemilt, & Jebb, 2015). To date, numerous studies have examined the impact that very large portion sizes have on energy intake and in such studies the amount of food served typically exceeds what a person can consume in a single sitting (Hollands et al., 2015; Zlatevska, Dubelaar, & Holden, 2014). However, less research has examined whether decreasing portion size reduces energy intake (Robinson & Kersbergen, 2018; Rolls, Roe, & Meengs, 2006) and in such studies the amount of food typically served is reduced from the amount of food served in commercially available food products (Rolls et al., 2006). Although it is plausible that people could compensate for reductions to portion size (e.g. eating more of other available food or increasing later energy intake), the limited number of studies conducted to date suggest that when main meal portion sizes are reduced energy intake tends to decrease (Lewis et al., 2015; Rolls et al., 2006). However, an important consideration when assessing the likely utility of this intervention approach at a population level is whether reducing portion size has a universal effect on energy intake (i.e. all people reduce their energy intake in response to smaller portions) or whether the benefits of reducing portion size are observed only among some population sub-groups.

The majority of research to date that has tested whether participant characteristics moderate the influence that portion size has on energy intake has examined the impact that large or very large portion sizes (as opposed to ‘normal’) have on eating behaviour. Results have been mixed. For example, BMI has been considered as a potential moderator of the influence of portion size in a number of studies and one study found that the influence of portion size was greater in participants with overweight and obesity than normal weight (Burger, Fisher, & Johnson, 2011). However, individual studies have often failed to find evidence that adults or children with overweight or obesity vs. normal weight differ in their response to larger portions (Kral, Remiker, Strutz, & Moore, 2014; Rolls et al., 2002; Zuraikat et al., 2018). Yet, contradicting these individuals studies, a meta-analysis by Zlatevska et al. found that the influence that larger portion sizes has on energy intake was larger among participants with normal weight compared to overweight or obesity (Zlatevska et al., 2014). Similarly, sex has been studied in relation to portion size and although numerous individual studies have found no support for moderation by sex (Rolls et al., 2002, 2007), some have (Robinson, te Raa, & Hardman, 2015). In addition, Zlatevska et al. reported that males show a stronger portion size effect than females in their meta-analysis (Zlatevska et al., 2014). However, a subsequent meta-analysis by Hollands et al. did not replicate the findings of Zlatevska et al., instead finding no evidence of moderation by BMI or sex (Hollands et al., 2015), although the Hollands et al. meta-analysis included other ‘size’ related manipulations (e.g. plate size) which may explain contradictory findings.

A range of traits have also been examined as potential moderators of the impact that portion size has on energy intake. Both dietary restraint and dietary disinhibition (also referred to as ‘external eating’) have been tested in multiple studies and no evidence of moderation has been found (Rolls et al., 2002; Zuraikat et al., 2018; Zuraikat et al., 2018). Likewise, in one study habitual plate clearing tendencies predicted how much energy a person consumed during a meal, but did not moderate the influence of portion size on energy intake (Sheen, Hardman, & Robinson, 2018). In a similar vein, concerns about wasting food were shown not to moderate the impact of larger portion sizes on energy intake in a different study (Zuraikat et al., 2018). Self-reported satiety responsiveness (the ability to regulate food intake in response to feelings of satiety) has been shown to moderate the influence of portion size on meal energy intake, whereby lower satiety responsiveness was associated with a larger impact of portion size on energy intake in children and in adults (Mooreville et al., 2015; Zuraikat et al., 2018). However, a subsequent study by the same research group did not replicate this finding in adults (Zuraikat et al., 2018).

Although these findings reveal potential moderators of the impact that large portions have on energy intake, they may not apply to the effects of reducing portion size. It has been suggested that humans may be more sensitive to the removal of energy from the diet (i.e. reducing portion size) than its addition (increasing portion size) as this may have been previously adaptive for survival (Blundell & Gillett, 2001). There may also be other differences in how consumers respond to very large portions vs. relatively small reduced portion sizes and the extent to which participant characteristics would be expected to explain responses. For example, an inability to regulate food intake in response to satiety (satiety responsiveness) may predict whether or not a person consumes large amounts of energy when it is readily available (i.e. when a very large portion has been served) but may be less important in explaining the impact that smaller portion sizes have on eating behaviour (Zuraikat et al., 2018). Likewise, dietary restraint has not been found to predict whether people consume more energy from larger vs. normal sized portions (Rolls et al., 2002; Zuraikat et al., 2018; Zuraikat et al., 2018, Zuraikat et al., 2018). Yet, it is feasible that smaller-reduced portion sizes may ‘license’ restrained eaters to increase their food intake of other available food more so than non-restrained eaters (Prinsen, Evers, & de Ridder, 2019; Sim, Lee, & Cheon, 2018), which would produce a differential effect of portion size on total energy intake based on individual differences in dietary restraint. With these considerations in mind, it is important to note that none of the studies conducted to date examining the effect of reducing portion size on energy intake have tested whether there are participant characteristics that moderate the impact of smaller portions on energy intake (French et al., 2014; Lewis et al., 2015; Rolls et al., 2006). It is therefore unclear whether there are participant characteristics that moderate the impact of reducing portion size on energy intake or whether reduced portion sizes decrease the energy intake of all.

In the present research we examined participant characteristic moderators of the impact that smaller portion sizes have on total energy intake by pooling data from three experimental studies of UK adults that used similar methodology to examine the impact that reducing the portion size served at a lunchtime meal has on total lunch energy consumed (Haynes, Hardman, Halford, Jebb, Mead, et al., 2020; Haynes, Hardman, Halford, Jebb, & Robinson, 2020). Motivated by the aforementioned research that has examined moderators of the effect that large portions have on energy intake, we explored participant sex, BMI, restrained eating, external eating (also sometimes referred to as ‘disinhibited eating’), plate clearing tendencies, food waste concerns and satiety responsiveness as potential moderators. For completeness we also examined the potential role of trait emotional eating, as although there is very mixed evidence, self-reported emotional eating shown to moderate the impact of environmental variables on energy intake (Bongers & Jansen, 2016). Likewise, we tested the moderating role of trait self-control, as there is some speculation that changes to the food environment that bypass the need for conscious intention (i.e. decreasing portion size) to promote healthier eating (i.e. reduced energy intake), may be particularly effective amongst groups of people who lack the self-control required to consistently limit their food intake (Marty, Jones, & Robinson, 2020). Because it is plausible that reducing portion sizes may cause consumers who are used to eating much larger portions to disregard smaller portion as being ‘appropriate’ or ‘normal’ (Peter Herman, Polivy, Pliner, & Vartanian, 2015; Robinson & Kersbergen, 2018), we also examined participants' usual portion size preferences as a potential moderator variable. Finally, as it is of public health relevance to understand whether most people respond to reductions to portion size as intended (i.e. decreased energy intake), we also examined the proportion of participants whose energy intake appeared to decrease when portion sizes were reduced (‘positive responders’), as opposed to showing no effect (‘non-responders’) or increase (‘negative responders’).

## 2 Methods

### 2.1 Overview of study design

The three studies we analyse data from have been reported in detail elsewhere; Study 1 and Study 2 (Haynes, Hardman, Halford, Jebb, & Robinson, 2020), Study 3 (Haynes et al., 2020). As part of each laboratory study participants were served lunchtime meals and the portion size of the meal differed based on session, resulting in lunchtime meal energy intake measurements under three portion size conditions (delivered in randomized order). The lunchtime portion sizes were selected based on portions identified as being ‘normal’ and ‘not normal’ in size by an independent sample of participants and during study piloting, also see (Haynes et al., 2019) for a detailed description. In the ‘Large-normal’ condition the portion sizes served were considered normal by most participants, in the ‘Small-normal’ condition the portion sizes were considered normal by most participants but approximately 75% of the size of the Large-normal condition, whereas in the ‘Smaller than normal’ condition portion sizes were considered smaller than normal by most participants and approximately 50% of the size of the Large-normal condition. In Study 1 and Study 3, if desired participants could serve themselves more of the same lunchtime food during the lunchtime session (buffet style from a large hot plate placed on a different table). In Study 2 participants could freely serve themselves dessert food after consuming the main meal portion size (buffet style placed on a different table). Thus, in each study we were able to examine the effect that varying the portion size of main meal food served impacted on total lunchtime energy intake. At the end of each study participants completed a battery of questionnaire measures that have not been analysed previously, in addition to a free-text response in which they were asked what they believe the aims of the study to be. Participants were financially reimbursed for their time and all studies were approved by the University of Liverpool research ethics committee.

### 2.2 Participants

Across all three studies eligibility criteria included no food allergies, intolerances or dietary requirements (e.g. gluten free, vegetarian), no history of eating disorders and willingness to consume the test foods in each study. In each study we recruited adults with a self-reported (screening questionnaire) BMI between 22.5 and 32.5 kg/m^2^ as the BMI of approximately 70% of adults in England fall within this range (NatCen Social Research, 2016) and approximately equal numbers of males and females and an equal number of participants in the two BMI bands characterising our eligibility criteria: 22.5–27.49 kg/m^2^ and 27.5–32.5 kg/m^2^. See Table 1 for individual study sample sizes.

**Table 1:** Participant characteristics across studies.

	Study 1	Study 2	Study 3	Pooled
	N = 45	N = 36	N = 30	N = 111
Age (years)	30.4 (SD = 12.8), 18-76	31.7 (SD = 11.9), 20-59	31.6 (SD = 10.3), 18-56	31.1 (SD = 11.8), 18-76
Sex	22 M, 23 F	18 M, 18 F	15 M, 15 F	55 M, 56 F
BMI	M = 27.3 (SD = 4.2), 18.9–42.2	M = 26.7 (SD = 3.6), 20.1–35.5	M = 26.1 (SD = 2.3), 22.5–29.8	M = 26.8 (SD = 3.6), 18.9–42.2
Usual portion size	M = 1.1 (SD = 0.4), 0.5–2.0	M = 0.9 (SD = 0.3), 0.4–1.8	M = 1.0 (SD = 0.2), 0.5–1.6	M = 1.0 (SD = 0.3), 0.4–2.0
Restrained eating	M = 2.5 (SD = 0.7), 1.0–4.0	M = 2.5 (SD = 0.8), 1.0–4.1	M = 2.6 (SD = 0.6), 1.4–3.5	M = 2.5 (SD = 0.7), 1.0–4.1
Emotional eating	M = 2.4 (SD = 1.0), 1.0–4.7	M = 2.4 (SD = 0.9), 1.0–4.5	M = 2.5 (SD = 0.9), 1.0–4.2	M = 2.4 (SD = 0.9), 1.0–4.7
External eating	M = 3.3 (SD = 0.7), 1.9–4.5	M = 3.2 (SD = 0.6), 2.0–4.5	M = 3.5 (SD = 0.6), 2.1–4.4	M = 3.3 (SD = 0.6), 1.9–4.5
Plate clearing tendencies	M = 4.3 (SD = 0.9), 1.6–5.0	M = 4.2 (SD = 0.9) 1.4–5.0	M = 4.5 (SD = 0.5), 3.0–5.0	M = 4.3 (SD = 0.8), 1.4–5.0
Food waste concerns	M = 4.9 (SD = 1.3), 2.2–7.0	M = 5.0 (SD = 1.2), 2.4–7.0	M = 5.5 (SD = 1.1), 2.8–7.0	M = 5.1 (SD = 1.2), 2.2–7.0
Self-control	M = 2.8 (SD = 0.8), 1.5–4.7	M = 3.1 (SD = 0.5), 2.0–4.1	M = 3.2 (SD = 0.5), 2.0–4.1	M = 3.0 (SD = 0.6), 1.5–4.7
Satiety responsiveness	–	–	M = 2.2 (SD = 0.6), 1.0–4.0	–

M = mean, SD = standard deviation, X-X = minimum and maximum values. BMI (body mass index) = measured weight (kg)/height (m)^2^. Usual portion size values are how participants' self-reported usual portion size correspond to manufacturer recommended serving size (e.g. 1.0 = same as recommended serving, 2.0 = twice the size of recommended serving). Restrained, Emotional and External eating are scored on 1–5 scales. Plate clearing tendencies are scored on a 1–5 scale, Food waste concerns are scored on a 1–7 scale. Self-control is scored on a 1–5 scale. Satiety responsiveness is scored on a 1–4 scale. Higher scores indicate increased tendencies for all measures.

### 2.3 Study foods

In Study 1, participants were served a portion of pasta with tomato sauce at lunchtime on three different days. In the Large-normal condition participants were served 336 g (307 kcal), in the Small-normal condition 252 g (230 kcal), and in the Smaller than normal 168 g (154 kcal). A serving bowl containing a further 200% of the recommended serving size of pasta and tomato sauce on a hot plate was in the room (on a different table) for participants to serve themselves more from if desired. In Study 2, participants were served a portion of chicken curry at lunchtime on three different days. In the Large-normal condition participants were served 423 g (506 kcal), in the Small-normal condition 325 g (389 kcal), and in the Smaller than normal condition 228 g (272 kcal). A dessert buffet containing bite-sized pieces of caramel shortbread (10 pieces, approx. 120 g, 575 kcal), and flapjack (10 pieces, approx. 150 g, 687 kcal) was also provided at lunch on a different table. The washout period between sessions in Study 1 and 2 was 7–10 days. In Study 3, participants attended ×3 five-day study session periods with a 1–6 week washout between study session periods. During each five-day study session period (Monday-Friday) the same ad-libitum breakfast was served, along with an ad-libitum snack box to consume from throughout the day. At lunchtime and dinner time the portion size of main meal food was manipulated. As Study 1 and Study 2 were conducted at lunchtime, for comparability we analyse data from the lunchtime sessions of Study 3. The lunchtime foods in Study 3 varied on a daily basis and were pesto penne pasta, spaghetti carbonara and spaghetti Bolognese. In the Large-normal condition the average energy content of served portion was 747 kcal, Small-normal (543 kcal) and Smaller than normal (339 kcals). The three types of lunch meals were served on rotation during the five-day study session periods and portion size order across ×3 five-day study session periods was randomly allocated. A serving bowl containing extra of the main meal food was on a hot plate on a different table for participants to serve themselves more from if desired. In each study, participants consumed the lunchtime meal individually in an eating behaviour laboratory. See online supplementary materials for number of participants consuming the initial serving in full and serving themselves additional food for each study.

### 2.4 Participant characteristic measures

#### 2.4.1 Sex

Participants self-reported their sex as part of a demographic questionnaire used to assess age and eligibility criteria.

#### *2.4.2* BMI

Weight and height were measured using a digital scale and stadiometer by a researcher in the laboratory. BMI was calculated by dividing weight (kg) by height (m)^2^.

#### 2.4.3 Restrained, emotional and external eating

Participants completed the Dutch Eating Behaviour Questionnaire (DEBQ; Van Strien, Frijters, Bergers, & Defares, 1986), which consists of 33 items in three subscales measuring restrained, external, and emotional eating tendencies. The scales have been demonstrated to have good internal reliability (Van Strien et al., 1986). Responses to 5-point Likert items were averaged to produce restrained, emotional and external eating scores ranging from 1 to 5 with higher scores indicating increased tendencies.

#### 2.4.4 Plate clearing tendencies

A 5-item scale was used to assess participants’ tendencies to clear their plate when eating (e.g., “I always clear my plate when eating”, 5-point Likert response option), items were averaged and higher scores indicate increased plate clearing tendencies. The scale has good internal reliability (Robinson, teRaa, & Hardman, 2015).

#### 2.4.5 Food waste concerns

Participants completed a self-devised 5-item self-report scale to assess attitudes toward food waste (e.g., “it is fine for food to go to waste sometimes”, 7-point Likert response option), with higher scores indicating greater concerns about wasting food.

#### 2.4.6 Self-control

The brief self-control scale was used to assess trait self-control (de Ridder, de Boer, Lugtig, Bakker, & van Hooft, 2011; Tangney, Baumeister, & Boone, 2004). Participants responded to 13-items on 5-point Likert scales ranging from 1 (strongly disagree), to 5 (strongly agree) (e.g., “I am good at resisting temptation”), with higher scores indicating greater self-control. The scale has good internal reliability (de Ridder et al., 2011; Tangney et al., 2004).

#### 2.4.7 Usual portion size

In Study 1, participants viewed images of the study food (pasta with tomato sauce) portions (ranging from 50% to 200% of the manufacturer's recommended serving size at 10% increment increases in portion size) as part of a computer-based questionnaire. The images were presented simultaneously and participants were asked to indicate which portion was closest to the amount of pasta with tomato sauce they would usually serve themselves. In Study 2 and 3, participants completed a computer-based task to indicate the portion size of the lunch meals served closest to their usual serving size. Participants adjusted the size of the displayed portion using the up and down arrow keys until it appeared equivalent to the amount of that food they would usually serve themselves. Each arrow key press increased or decreased the portion by an increment of 10% of the recommended serving, from a minimum of 40% to a maximum of 300%. Because of the difference in measurement tool across studies we Z-scored usual portion ratings for each study for analysis purposes. Higher scores indicate a larger usual portion.

#### 2.4.8 Satiety responsiveness

In Study 3 only, participants completed the 4-item satiety responsiveness subscale of the Adult Eating Behaviour Questionnaire (e.g., “I often get full before my meal is finished”) (Hunot et al., 2016), in which they indicated their agreement with each statement on a 5-point Likert response scale ranging from 1 (strongly disagree) to 5 (strongly agree), with higher scores indicating greater satiety responsiveness. The scale has been validated as a measure of appetitive traits in adults and has good internal consistency (Hunot et al., 2016).

### 2.5 Analysis strategy

#### 2.5.1 Participant characteristics and moderation

We pre-registered our planned analyses after data collection and submission of studies 1–3 for publication, but prior to pooling participant characteristic data in order to conduct the present research, see https://osf.io/gm7ne/. We planned to restrict analyses to participants with complete data on all portion size sessions within their respective randomized controlled trial and the participant characteristic variables described above. In our primary analyses we conducted a series of 10 mixed ANOVAs (dependent variable = total lunchtime energy intake for Study 1 and Study 2, and in Study 3 total lunchtime energy intake averaged across 5 sessions) for each proposed moderator variable, with a between-subjects factor of Study (Study 1, Study 2, Study 3), within-subjects factor of Portion Size Condition (Large-normal, Small-normal, Smaller than normal) and the participant characteristic measure as a covariate. To correct for multiple comparisons the criterion for statistical significance was adjusted to p < .005 (0.05/10 hypothesised moderators) in these models. If there was evidence of a significant Portion Size Condition × Moderator interaction for any of the moderators, we planned to use the macro ‘MEMORE’ for SPSS to investigate the direction of the interaction (Montoya, 2019). In each study, participants reported what they believed were the aims of the study. Two coders independently assessed participant free text responses to determine awareness of the influence of portion size on their eating/appetite when asked to guess the study aims. We planned to repeat the primary analyses with these participants removed. We also planned sensitivity analysis with any participants who were outliers on total lunchtime energy intake (outlier status determined on a study-by-study basis, identified as those with a value >3SD from condition mean).

#### 2.5.2 Statistical power for participant characteristic moderation analysis

Cohen's f ANOVA effect size guidelines are 0.1 = small, 0.25 = medium, 0.4 = large. To examine a between-within subjects interaction (portion size × moderator variable interaction) in a mixed ANOVA, a sample size of ~100 participants provides adequate statistical power to detect a relatively small sized effect of f = 0.13 in our main analyses (80% power, p < .005, r~0.7 for within-subjects correlation). Based on our main analysis sample size (n = 111) using the above parameters we were powered to detect interaction effects of f = 0.12 and greater in ANOVA models.

#### 2.5.3 Responder status

We examined the proportion of participants in each study that ‘responded’ by eating less (overall energy intake, including initial serving and additional food) when served a smaller vs. larger portion size. We used a 10% difference cut-off to determine ‘responder’ status as we were aware of no validated cut-off level, although a difference of 10% has been suggested to be of practical relevance in appetite research (Blundell et al., 2010). Our main planned contrast was between the Large-normal portion size condition and Smaller than normal portion size condition (most extreme portion size comparison) and any participant whose energy intake measured in their Smaller than normal portion size trial was at least 10% less than their measured energy intake in their Large-normal portion size condition was classed as a ‘positive responder’. Conversely, participants with an energy intake reduction of between −9.9 and + 9.9% in the smaller relative to the larger portion size condition were classed as ‘non-responders’ and participants who showed an increase of at least 10% were classed as ‘negative responders’. In addition, to examine consistency of responder status we calculated the number of participants who were consistently categorised across all portion size contrasts (e.g. number of people who were ‘positive responders’ in all contrasts). We also repeated the above procedure using a 5% rather than 10% cut-off to determine responder status in order to examine if results were consistent.

## 3 Results

### 3.1 Participant characteristics

Across the three studies, complete data was available for 111 participants (55 male, 56 female), with a mean age of 31.1 (SD = 11.8) years. The sample had a mean BMI of 26.8 (SD = 3.6), with 40 participants classed as normal weight (BMI 18.5–24.9), 55 classed as having overweight (BMI 25–29.9) and 16 participants classed as having obesity (BMI ≥ 30). See Table 1 for participant characteristics.

### 3.2 Effect of portion size on energy intake

In an ANOVA including no participant characteristic moderator variables (N = 111), there was a significant main effect of Study Origin [F = 64.28, ηp^2^ = 0.54, p < .001], significant main effect of Portion Size [F = 54.01, ηp^2^ = 0.33, p < .001] and no significant interaction between Portion Size and Study Origin [F = 1.46, ηp^2^ = 0.0.03, p = .22]. The main effect of Study Origin was explained by there being a lower average energy intake in Study 1 (M kcals consumed = 310.7, SE = 26.0) compared to Study 2 (M kcals consumed = 682.6, SE = 29.1, p < .001) and Study 3 (M kcals consumed = 705.5, SE = 31.9, p < .001). There was no significant difference in intake between Study 2 and 3 (p = .60). For the main effect of Portion Size, energy intake was significantly reduced as portion size decreased from Large-normal (estimated marginal M kcals consumed = 620.4, SE = 18.2), to Small-normal (estimated marginal M kcals consumed = 563.7, SE = 17.2) to Smaller than normal (estimated marginal M kcals consumed = 514.7, SE = 17.9) and all portion size conditions significantly differed from each other (p < .001).

### 3.3 Moderation of portion size effect on energy intake by participant characteristics

In primary analyses (N = 111), there was no evidence of significant moderation by any of the participant characteristics. See Table 2. In sensitivity analyses in which participants who were outliers on energy intake or guessed the aims of the study were removed (N = 18) results were largely unchanged. The only change of note was that the interaction between Portion Size and BMI became closer to statistical significance [F = 4.31, ηp^2^ = 0.05, p = .02], but was not statistically significant based on our a-priori alpha level adjusted for multiple comparisons (p = .005). For descriptive purposes we probed this marginally significant interaction (Supplementary File 1, Table S1). The effect of reducing portion size to ‘Smaller than normal’ from ‘Small-normal’ had a significant effect on energy intake among individuals with higher BMI but not those with lower BMI. However, the effect of reducing portion size to ‘Small-normal’ from ‘Large-normal’ had a slightly stronger effect on energy intake among individual with lower BMI than those with higher BMI, although the effect of portion was still statistically significant for both ‘lower’ and ‘higher’ BMI groups across both comparisons. There was no evidence that the effect of portion size on energy intake was moderated by BMI for the most extreme portion size contrast (reduction to ‘Smaller than normal’ from ‘Large-normal’). See Supplementary File 1, Table S1.

**Table 2:** Results of ANOVA models examining moderation of portion size by participant characteristics controlling for study origin.

	Main effect of Study origin	Main effect of Portion size	Main effect of participant characteristic factor	Portion size × participant characteristic factor interaction
Sex	df = 2, F = 74.08, ηp^2^ = .581, p < .001	df = 2, F = 7.11, ηp^2^ = .062, p = .001	df = 1, F = 18.44, ηp^2^ = .147, p < .001	df = 2, F = 0.13, ηp^2^ = .001, p = .88
BMI	df = 2, F = 61.88, ηp^2^ = .536, p < .001	df = 2, F = 3.36, ηp^2^ = .030, p = .04	df = 1, F = 1.80, ηp^2^ = .017, p = .18	df = 2, F = 2.74, ηp^2^ = .025, p = .07
Usual portion size	df = 2, F = 83.64, ηp^2^ = .610, p < .001	df = 2, F = 53,73, ηp^2^ = .033, p < .001	df = 1, F = 30.04, ηp^2^ = .219, p < .001	df = 2, F = 0.49, ηp^2^ = .005, p = .61
Restrained eating	df = 2, F = 64.55, ηp^2^ = .547, p < .001	df = 2, F = 1.12, ηp^2^ = .010, p = .33	df = 1, F = 0.79, ηp^2^ = .007, p = .38	df = 2, F = 0.95, ηp^2^ = .009, p = .39
Emotional eating	df = 2, F = 63.19, ηp^2^ = .542, p < .001	df = 2, F = 7.63, ηp^2^ = .067, p = .001	df = 1, F = 0.47, ηp^2^ = .004, p = .50	df = 2, F = 0.45, ηp^2^ = .004, p = .64
External eating	df = 2, F = 63.50, ηp^2^ = .543, p < .001	df = 2, F = 1.03, ηp^2^ = .010, p = .36	df = 1, F = 0.00, ηp^2^ = .000, p = .98	df = 2, F = 0.14, ηp^2^ = .001, p = .87
Plate clearing tendencies	df = 2, F = 70.00, ηp^2^ = .556, p < .001	df = 2, F = 2.06, ηp^2^ = .019, p = .13	df = 1, F = 6.90, ηp^2^ = .061, p = .01	df = 2, F = 0.99, ηp^2^ = .009, p = .37
Food waste concerns	df = 2, F = 61.19, ηp^2^ = .534, p < .001	df = 2, F = 0.33, ηp^2^ = .003, p = .72	df = 1, F = 3.18, ηp^2^ = .029, p = .08	df = 2, F = 1.31, ηp^2^ = .012, p = .27
Self-control	df = 2, F = 59.69, ηp^2^ = .527, p < .001	df = 2, F = 2.06, ηp^2^ = .019, p = .13	df = 1, F = 0.01, ηp^2^ = .000, p = .92	df = 2, F = 0.08, ηp^2^ = .001, p = .93
Satiety responsiveness	–	df = 2, F = 1.33, ηp^2^ = .045, p = .27	df = 1, F = 0.45, ηp^2^ = .016, p = .51	df = 2, F = 0.55, ηp^2^ = .019, p = .55

N = 111. Raw data for participant characteristic factors was used in all analyses, with the exception of usual portion size (z-scored in each study to account for slight measurement differences across studies).

### 3.4 Unplanned moderation analyses

We did not originally plan to test participant age as a potential moderator variable, but as inspection of pooled data indicated reasonable variation in age (M = 31.1 yrs, SD = 11.8, 60% of sample = 18–29yrs old, 40% of sample = 30 years and older) we conducted an exploratory moderation analysis. There was no evidence in the full (N = 111, ηp^2^ = 0.009, p = .37) or reduced (N = 93, ηp^2^ = 0.006, p = .57) sample that age moderated the effect of portion size on energy intake. At the request of an anonymous peer reviewer, we also re-ran the moderation of portion size effect on energy intake by participant characteristic factors replacing the dependent variable (total energy consumed) with percentage of energy consumed relative to the amount of energy consumed in the Large-normal portion size condition. This approach accounts for differences in energy consumed across the three studies. Results were the same as in the main analysis (no significant moderation), with the exception of BMI. As in the results of the sensitivity analyses described above, there was some evidence of moderation by BMI. However, BMI only moderated the effect of reducing portion size to ‘Smaller than normal’ from ‘Small-normal’: this reduction had a significant effect on intake as a percentage relative to intake in the Large normal portion size condition among individuals with higher BMI but not those with lower BMI. There was no evidence that the effect of portion size on energy intake (%) was moderated by BMI for either of the other portion size contrasts. See online supplementary materials for results in full.

### 3.5 Characterising portion size ‘responder’ status

In the most extreme portion size contrast (‘Large-normal’ condition vs. ‘Smaller than normal’ condition), 72% of participants across studies reduced their energy intake by 10% or more (‘positive responders’) when portion size was reduced from the larger to smaller portion size. Of the remaining participants, 20% were ‘non-responders’ (energy intake in the smaller portion size condition was within −9.9 to +9.9% of energy intake in the larger portion size condition) and 8% were ‘negative responders’ (energy intake in the smaller portion size condition was ≥10% of energy intake in the larger portion size condition). See Table 3. For the less extreme portion size contrasts, the average number of ‘positive responders’ was 51%, with 32% ‘non-responders’ and 17–18% ‘negative responders’. Examining consistency of responder classification across studies, it was most common (69%) for participants to have inconsistent responder classification across the three portion size contrasts (e.g. being a ‘positive’ responder in two contrasts, but a ‘non-responder’ in one contrast). After this the next most common classification was ‘consistent positive responder’ (23%), whereas few participants were consistent ‘non-responders’ (6%) or ‘negative responders’ (1%). See Table 4. Characterising responder status using a 5% difference cut off (as opposed to 10% as described above) produced the same pattern of results for individual portion size contrast responder classifications (i.e. the majority of participants were classed as ‘positive responders’) and consistency of responder classification (inconsistent responder classification was most common, followed by consistent positive responder), as reported in the online supplemental materials. See Supplementary File 1, Tables S3–S4.

**Table 3:** Summary of participants classified as positive, non and negative responders to portion size reductions.

	Large-normal vs. smaller than normal portion size			Large-normal vs. small-normal portion size			Small-normal vs. smaller than normal portion size		
	+ve responder	Non responder	-ve responder	+ve responder	Non responder	-ve responder	+ve responder	Non responder	-ve responder
Study 1 (n = 45)	33 (73%)	8 (18%)	4 (9%)	26 (58%)	9 (20%)	10 (22%)	24 (53%)	11 (24%)	10 (22%)
Study 2 (n = 36)	26 (72%	6 (17%)	4 (11%)	15 (42%)	14 (39%)	7 (19%)	21 (58%)	10 (28%)	5 (14%)
Study 3 (n = 30)	21 (70%)	8 (27%)	1 (3%)	15 (50%)	12 (40%)	3 (10%)	12 (40%)	14 (47%)	4 (13%)
Across studies (n = 111)	80 (72%)	22 (20%)	9 (8%)	56 (51%)	35 (32%)	20 (18%)	57 (51%)	35 (32%)	19 (17%)

+ve responder refers to number of participants whose energy intake was reduced by 10% or more when comparing energy intake in the larger portion size condition vs. smaller portion size condition of contrast (eating less in smaller portion condition of contrast). Non-responder refers to number of participants who energy intake was between +9.9% and −9.9% when comparing energy intake in the larger portion size condition vs. smaller portion size condition of contrast. –ve responder refers to number of participants whose energy intake was increased by 10% or more when comparing energy intake in the larger portion size condition vs. smaller portion size condition of contrast (eating more in smaller portion condition of contrast).

**Table 4:** Summary of participants consistently classified as positive, non and negative responders to portion size reductions across all portion size contrasts.

	Consistently classed as + ve responder	Consistently classed as non-responder	Consistently classed as –ve responder	Inconsistent responder classification
Study 1 (n = 45)	13 (29%)	3 (7%)	1 (2%)	28 (62%)
Study 2 (n = 36)	7 (19%)	0 (0%)	0 (0%)	29 (81%)
Study 3 (n = 30)	6 (20%)	4 (13%)	0 (0%)	20 (67%)
Across studies (n = 111)	26 (23%)	7 (6%)	1 (1%)	77 (69%)

‘Consistently classed’ indicates participant was a +ve, non or –ve responder across all 3 portion size reduction contrasts (i.e. large-normal vs. smaller than normal, large-normal vs. small-normal, small-normal vs. smaller than normal). Inconsistent classification indicates number of participants whose responder classification was not the same across all 3 portion size reduction contrasts.

## 4 Discussion

In the present research we examined potential participant characteristic moderators of the effect that reducing portion size has on main meal energy intake. We found no convincing evidence that any studied participant characteristics significantly moderated the effect portion size had on energy intake. These findings are consistent with the notion that most a people consume less energy when main meal portion size is reduced.

Although we failed to find convincing evidence of significant moderation from the studied participant characteristics, it is still plausible that specific sub-groups of participants do not decrease their energy intake when portion size is reduced. Therefore, another aim of the present research was to quantify the proportion of participants who appeared to decrease their meal energy intake when portion size was decreased. We explored this by examining how common it was for participant energy intake to reduce when comparing their energy intake from a larger vs. smaller portion. For the most extreme portion size comparison (when the smaller portion size was ~50% of the larger portion), the majority of participants consumed less energy. We also observed that for a minority of participants, energy intake did not decrease or, counter-intuitively, it increased. This is to be expected due to random error and daily fluctuations in appetite, as energy intake for each portion size was measured only once (in studies 1 and 2, and over 5 days in study 3) and there is evidence that energy intake can vary greatly on a day-to-day basis (Levitsky et al., 2017). In support of this, only for very few participants did energy intake consistently (i.e. across multiple portion size contrasts) increase or remain the same when portion size was decreased. Instead it was most common for participants to show an inconsistent response to portion size reductions (e.g. reduced energy intake when comparing two of the contrasts, but no reduction for the third contrast). Again, we presume this is in part explained by random error and variations in daily appetite. Yet, for the majority of participants energy intake decreased when portion size was reduced and it was more common for participants to consistently reduce their energy intake than consume a similar amount when portion size was reduced. Collectively these findings suggest that portion size is likely to impact on meal energy intake for most people and reducing food portion sizes may be an equitable public health approach to reducing population level energy intake.

The methodology used in the present research differs to traditional portion size studies. Typically portion size studies have provided portion sizes in both the ‘normal’ and ‘large’ portion size conditions so large that participants are unable to finish them, in part to prevent ceiling effects on food intake (Rolls et al., 2002). However, in real world settings portion sizes will be smaller and food served is often eaten in its entirety (Fay et al., 2011). In the present studies participants were served initial portion sizes that would be more representative of the amount of food served in the real world (i.e. the portion sizes were selected based on how normal their size appeared), but critically extra food was readily available to participants and this still allowed participants to ‘compensate’ for smaller portions by eating more after their initial serving. This meant that it was fairly common for participants to consume the initial portion served in full, which is typically not the case in previous portion size research and highlights how the methodological paradigm used in the present studies differs markedly to studies examining the impact of large portion sizes on energy intake. In addition to our focus on smaller portion sizes, this methodological difference may also account for why the present results are not at times consistent with other portion size literature. For example, Zlatevska and colleagues found that males were more influenced by larger portion sizes than females in their meta-analysis (Zlatevska et al., 2014), but we found no such evidence.

We examined a range of potential demographic, psychological and behavioural individual difference moderators of the effect of portion size, but it may be the case that there are important moderators yet to be identified. In addition, satiety responsiveness was measured in only one study. Because there is some mixed evidence that satiety responsiveness moderates the effect of large portions on energy intake in both children and adults (Kral et al., 2014; Mooreville et al., 2015; Zuraikat et al., 2018; Zuraikat et al., 2018), further research may benefit from examining it as a potential moderator of the effect of smaller portions on energy intake. A number of the measurement tools we used to measure participant characteristic moderators have been adopted in previous research examining portion size, such as the adult eating behaviour questionnaire to measure satiety responsiveness (Zuraikat et al., 2018) and the plate clearing tendencies scale (Sheen et al., 2018), although a number have not been used to date (e.g. food waste concerns) and therefore further research examining these lesser studied factors may be informative. A further proposed moderator of the influence of portion size on energy intake is food liking/palatability (Zuraikat et al., 2018). We did not test this in the present analysis because well-liked foods were selected for use and a participant eligibility criterion for studies was liking the test foods used, which minimises variability in liking ratings. The recruitment strategy used in these studies meant that we had an even number of males and females, as well as a stratified sample based on BMI. There were relatively few participants with obesity (14%), so it may be the case that results would have been different had we sampled more participants from the obese BMI range.

Strengths of the present research include the use of pooled data from three controlled trials that used similar methodology to measure meal time energy intake, which provided statistical power to detect relatively small moderation effects. Although there were some methodological differences between studies and this is a limitation of the present research (e.g. the food used in Study 1 was less calorific compared to Study 2 and 3), our analyses controlled for between study differences in energy intake and there was no evidence that any effects were dependent on study (i.e. there was no evidence that the effect of portion size on energy intake was moderated by study). However, it would be more robust to examine evidence for moderation across studies using the exact same methodology or to have a larger total sample size when studies with differing methodologies are combined. A limitation to the scope of the present research was that we were unable to examine the importance of socioeconomic position (SEP). SEP has been linked to portion size preferences and was recently shown to moderate the impact that larger portion sizes had on hypothetical intended consumptions in an online study, whereby participants of lower SEP were more influenced by portion size (Best & Papies, 2019). It is therefore plausible that the effect of reducing portion size on energy intake may differ based on SEP and given that reducing portion sizes is a potential public health approach to improve diet, future research would benefit from considering SEP.

Like the majority of research on portion size to date (Hollands et al., 2015), the present findings tell us about the effect of portion size on acute meal energy intake and do not speak to the prolonged effects of reducing portion size. It may be the case that some factors would predict whether or not a person adjusts their energy intake over a longer period in response to reduced portion sizes and future research will be required to address this. Finally, the present findings relate to adults’ responses to portion size. The results of a recent meta-analysis demonstrated that larger portion size increase intake among children aged 2–12 years, and that neither child age, food type (unit-based or amorphous), nor the size of the baseline portion moderate this effect (Reale et al., 2019). Further research on whether children consistently respond to *reduced* portion sizes of food and whether individual eating traits such as satiety or food responsiveness moderate these responses would be useful to inform an understanding of the universality of portion size effects in children.

## 5 Conclusions

Portion size may be a universal driver of energy intake, as reducing meal portion size appears to decrease meal energy intake among most people. Food portion downsizing may therefore be an equitable intervention approach to reducing population level energy intake.

## Availability of data and materials

The study dataset and registered protocol is available on the Open Science Framework repository at https://osf.io/gm7ne/.

## Funding

This research was supported by a NIRG awarded by the 10.13039/501100000265Medical Research Council (10.13039/501100000265MRC) to ER (MR/N00218/1). The funding body had no role in study design, data collection and analysis, decision to publish, or preparation of the manuscript. ER's salary was supported by the 10.13039/501100000265MRC.

## Author contributions

ER and AH contributed to designing the research and analysed the data. ER drafted the manuscript, and AH contributed to the final written manuscript. Both authors approved the final manuscript.

## Declaration of competing interest

ER has previously received research funding from the 10.13039/100012228American Beverage Association and 10.13039/100007190Unilever for projects unrelated to the present work.
