# Supplementary material for: Individual differences and moderating participant characteristics in the effect of reducing portion size on meal energy intake: Pooled analysis of three randomized controlled trials
Source: Appetite. 2021 Apr 1;159:105047. doi: 10.1016/j.appet.2020.105047 (PMC7816161; doi:10.1016/j.appet.2020.105047)
Supplement: Multimedia component 1 [file mmc1.docx]

**Online Supplementary Materials**

**Participants consuming all of initial portion in studies**

To account for measurement error, participants consuming > 95% of the initial portion (calculated by comparing the kilocalories consumed from the initial portion [pre-meal - post-meal food weight * calorie density per gram] to the kilocalories served to participants in the initial portion) were considered plate clearers. Averaged across studies, in the Smaller than normal condition 93.8% of participants were plate clearers, 79% in the Small-normal condition and 71.6% in the Large-normal condition. We note that it was not possible at the time of the study to determine whether any food remaining on participants’ plates in Studies 1 or 3 was left-over from the initial portion or was additional food served by the participants but not eaten (as the additional food was the same as the initial served food). This allows for the possibility that some participants could be misclassified as non-plate clearers (of the initial portion). However, of all the participants who were classified as non-plate clearers across conditions, none were classed as having served themselves additional food suggesting that being a plate clearer was a logical pre-requisite for being classed as serving additional food (see below).

**Participants serving themselves additional food in studies**

To account for measurement error, participants serving themselves > 5% of the additional food available (calculated by comparing the kilocalories self-served by participants from the additional food provided [pre-meal weight – post-meal food weight * calorie density per gram] to the kilocalories in the additional food made available to participants) were considered to have served themselves a notable amount of additional food. Averaged across studies, in the Smaller than normal condition this number was 81.5%, 70.4% in the Small-normal condition and 61.7% in the Large-normal condition. In Study 2, 33.3% of participants who were classed as non-plate clearers consumed additional food (classed as ‘serving themselves additional food’ here), which is explained by the additional food being a different type of sweet dessert food (which participants would want consume even if they had not finished their initial portion of the other main meal food). In Studies 1 and 3, because the initial portion and additional food available was the same type of food it was not possible to conclusively determine at the time of the study whether all additional food served by participants was consumed. However, all participants in Studies 1 and 3 who were classed as having ‘served themselves additional food’ were also classed as ‘plate clearers’, suggesting that all or most of additionally served food was consumed.

**BMI Moderation Follow Up Analyses (from sensitivity analyses)**

We used the macro MEMORE for SPSS to investigate the direction of the interaction between portion size and BMI. A separate moderation model was conducted for each pairwise portion size contrast controlling for study (dummy coded). Estimates of the effect of portion size on intake was estimated at +1 and -1SD from the mean BMI for the ‘Smaller than normal’ versus ‘Small-normal’ and for the ‘Small-normal’ versus ‘Large-normal’ models, as these were the comparisons in which BMI was associated with the size of the portion effect. See Table S1 below. Reducing portion size to ‘Smaller than normal’ from ‘Small normal’ had a significant effect on energy intake among individuals with ‘higher’ BMI but not those with ‘lower’ BMI, however reducing the portion size to ‘Small-normal’ from ‘Large-normal’ had a stronger effect on energy intake among individuals with ‘low’ BMI than those with ‘high’ BMI.

Table S1. BMI moderation results

|  | Effect of BMI on portion size effect | Effect of portion size reduction by BMI^a^ | |
| --- | --- | --- | --- |
|  |  | Low BMI (*M*-1*SD*) | High BMI (*M*-1*SD*) |
| Smaller than normal versus Small-normal | -7.19 (2.88)* | -23.66 (14.34) | -74.57 (14.34)*** |
| Small-normal versus Large-normal | 7.45 (2.64)** | -91.71 (13.38)*** | -35.81 (13.38)** |
| Smaller than normal versus Large-normal | 0.26 (3.11) | - | - |

**p* <.05, ***p*<.01, ****p*<.001. Note. Values are *B*(*SE*). ^a^ Models reported are run across studies (rather than controlling for study origin).

**Unplanned Moderation Analyses**

At the request of an anonymous peer reviewer, we also re-ran the moderation of portion size effect on energy intake by participant characteristics replacing the dependent variable (total energy consumed) with percentage of energy consumed relative to the amount of energy consumed in the Large-normal portion size condition. In a model including only study origin and portion size condition there was no significant main effect of Study Origin [F=1.039, p = .36], a significant main effect of Portion Size [F=39.41, p<.001] and no significant interaction between Portion Size and Study Origin [F=1.20, = .31]. The main effect of Portion Size was explained by a decrease in % consumed relative to energy intake in the ‘Large-normal’ condition (estimated marginal M = 100%) vs. ‘Small-normal’ condition (estimated marginal M = 92.0%) vs. ‘Smaller than normal’ condition (estimated marginal M = 82.3%). There was no evidence of significant moderation by participant characteristics, with the exception of BMI. In the model examining BMI, there was a significant main effect of portion size condition (df=2, F=6.28, p=.002), no significant main effect of study origin (df=2, F=1.18, p=.21) or BMI (df=1, F=1.09 p=.299) and a significant portion size condition*BMI interaction (df=2, F=7.02, p=.001).

We next used the macro MEMORE for SPSS to investigate the direction of the interaction between portion size and BMI. A separate moderation model was conducted for each pairwise portion size contrast controlling for study (dummy coded). Estimates of the effect of portion size on intake as a % of intake relative to intake in the ‘Large-normal’ condition was estimated at +1 and -1SD from the mean BMI for the ‘Smaller than normal’ versus ‘Small-normal’ model, as this was the only comparisons in which BMI was associated with the size of the portion effect. See Table S2 below. Reducing portion size to ‘Smaller than normal’ from ‘Small normal’ had a significant effect on intake as a % of intake relative to intake in the ‘Large-normal’ condition among individuals with ‘higher’ BMI but not those with ‘lower’ BMI.

Table S2. BMI moderation results

|  | Effect of BMI on portion size effect | Effect of portion size reduction by BMI^a^ | |
| --- | --- | --- | --- |
|  |  | Low BMI (*M*-1*SD*) | High BMI (*M*-1*SD*) |
| Smaller than normal versus Small-normal | -1.94 (0.55)*** | -2.95 (2.75) | -17.61 (2.75)*** |
| Small-normal versus Large-normal | 1.45 (0.55) | - | - |
| Smaller than normal versus Large-normal | -0.49 (0.52) | - | - |

**p* <.05, ***p*<.01, ****p*<.001. Note. Values are *B*(*SE*). ^a^ Models reported are run across studies (rather than controlling for study origin).

|  | Large-normal vs. smaller than normal portion size | | | Large-normal vs. small-normal portion size | | | Small-normal vs. smaller than normal portion size | | |
| --- | --- | --- | --- | --- | --- | --- | --- | --- | --- |
|  | +ve responder | Non responder | -ve responder | +ve responder | Non responder | -ve responder | +ve responder | Non responder | -ve responder |
| Study 1 (n=45) | 36 (80%) | 5 (11%) | 4 (9%) | 30 (67%) | 4 (9%) | 11 (24%) | 27 (60%) | 7 (16%) | 11 (24%) |
| Study 2 (n=36) | 26 (72% | 4 (11%) | 6 (17%) | 21 (58%) | 7 (19%) | 8 (22%) | 24 (67%) | 5 (14%) | 7 (19%) |
| Study 3 (n=30) | 21 (70%) | 5 (17%) | 4 (13%) | 21 (70%) | 5 (17%) | 4 (13%) | 19 (63%) | 6 (20%) | 5 (17%) |
| Across studies (n=111) | 83 (75%) | 14 (13%) | 14 (13%) | 72 (65%) | 16 (14%) | 23 (21%) | 70 (63%) | 18 (16%) | 23 (21%) |

Table S3. Summary of participants classified as positive, non and negative responders to portion size reductions (5% difference)

+ve responder refers to number of participants whose energy intake was reduced by 5% or more when comparing energy intake in the larger portion size condition vs. smaller portion size condition of contrast (eating less in smaller portion condition of contrast). None responder refers to number of participants who energy intake was between +4.9% and -4.9% when comparing energy intake in the larger portion size condition vs. smaller portion size condition of contrast. –ve responder refers to number of participants whose energy intake was increased by 5% or more when comparing energy intake in the larger portion size condition vs. smaller portion size condition of contrast (eating more in smaller portion condition of contrast).

Table S4. Summary of participants consistently classified as positive, non and negative responders to portion size reductions across all portion size contrasts (5% difference)

|  | Consistently classed as +ve responder | Consistently classed as non-responder | Consistently classed as –ve responder | Inconsistent responder classification |
| --- | --- | --- | --- | --- |
| Study 1 (n=45) | 15 (33%) | 1 (2%) | 1 (2%) | 28 (62%) |
| Study 2 (n=36) | 10 (28%) | 0 (0%) | 1 (3%) | 25 (69%) |
| Study 3 (n=30) | 14 (47%) | 3 (10%) | 1 (3%) | 12 (40%) |
| Across studies (n=111) | 39 (35%) | 4 (4%) | 3 (3%) | 65 (59%) |

‘Consistently classed’ indicates participant was a +ve, non or –ve responder across all 3 portion size reduction contrasts (i.e. Large-normal vs. smaller than normal, large-normal vs. small-normal, small-normal vs. smaller than normal). Inconsistent classification indicates number of participants whose responder classification was not the same across all 3 portion size reduction contrasts.
